# The Fourth Annual Commencement of the Maryland Dental College

**Published:** 1877-04

**Authors:** 


### ARTICLE V.

*The Fourth Annual Commencement of the Maryland Dental College, of Baltimore City*, was held at the Academy of Music, on the evening of the first day of March.

The Rev. F. Swentzell, M. D., opened the exercises with prayer.

After an appropriate address to the graduating class, Dr. R. B. Donaldson, of Washington, D. C., President of the Board of Regents, conferred the degree of Doctor of Dental Surgery upon the following gentlemen:

| | |
|---|---|
| Preston A. Ames, - | Maryland. |
| B. F. Barclay. - - | Pennsylvania. |
| John D. Clark, - - | North Carolina. |
| H. Olie Duerson, - | Virginia. |
| George D. Fouke, - - | Maryland. |
| Cevileon Fones, - - | Connecticut. |
| Henry A. Gaylord, - | Massachusetts. |
| P. L. Hull, - - | New York. |
| Ernest F. King, - - | Maryland. |
| A. P. Krouse, - - | Maryland, |
| Wm. B. Mann, - - | Maryland. |
| Geo. S. Meigs, - - | New York. |
| Chas. J. Peterson, - | Iowa. |
| Frank H. Paign, - - | New York. |
| A. W. Ross, - | South Carolina. |
| J. B. L. Swentzell, - | Maryland. |
| J. B. Thresher, - - | Connecticut. |

The Annual Address was then delivered by Dr. Jesse C. Green, one of the Regents, and was followed by the Class Valedictory Address of A. W. Ross, of South Carolina, which was highly appreciated and applauded.

After the more important exercises had closed with a benediction by the Rev. S. D. Noyes, the Annual Alumni Hop took place; the beauty of Baltimore was well represented, and the "light fantastic toe" was tipped to heart's content in the mazy dance, and all went merrily as "marriage bells."

